# Self-disclosure and relational agents for mental health: a scoping review protocol

**DOI:** 10.1136/bmjopen-2025-100613

**Published:** 2025-08-24

**Authors:** Sia Sha, Kate Loveys, Isla Francis, Eric Ji, Leo Lyu, Dario Krpan, Matteo Maria Galizzi, Mark Taylor

**Affiliations:** 1 The London School of Economics and Political Science, London, UK; 2 The University of Auckland, Auckland, New Zealand; 3 Goldsmiths University of London, London, UK

**Keywords:** Public health, MENTAL HEALTH, Health informatics

## Abstract

**ABSTRACT:**

**Introduction:**

Relational agents are an innovative form of Artificial Intelligence (AI) that can help address waiting times for mental health services. They often appear in the form of chatbots that provide responses to patient questions via web or mobile interfaces, and they seek to build long-term relationships with patients. Effective self-disclosure is key for therapeutic outcomes, and we are therefore conducting a scoping review to map the literature on self-disclosure to relational agents for mental health.

**Methods:**

Our work will follow guidance by the Joanna Briggs Institute and the Preferred Reporting Items for Systematic Reviews and Meta-Analyses (PRISMA) Extension for Scoping Reviews. We will systematically search Ovid Medline, Ovid Embase, Ovid Emcare, Ovid PsycINFO, Ovid Global Health, EBSCO CINAHL, Scopus, Web of Science and ProQuest Dissertations & Theses Global. Two reviewers will independently screen titles and abstracts as well as full texts of potential studies in Covidence. Both qualitative and quantitative studies from all countries published in English will be eligible. We will then provide a narrative synthesis of the results along with data tables.

**Ethics and dissemination:**

Our scoping review does not require ethical approval. We will publish results in a peer-reviewed journal and during conference presentations.

**Trial registration number:**

Open Science Framework (https://osf.io/wf4aq).

**STRENGTHS AND LIMITATIONS OF THIS STUDY**

- The scoping review comprehensively searches 9 databases without restriction on publication date.
- The review focuses on English studies only.
- Both qualitative and quantitative studies from all countries are eligible.

## Introduction

 The National Heatlh Service (NHS) faces significant challenges in its mental health provision. There is a shortage of staff, and turnover for staff occurs at a higher rate than elsewhere in the NHS due to a variety of issues such as lower job satisfaction.^1^ Patient care is directly impacted: while the NHS aims for patients to receive treatment within 18 weeks of referral to its mental health services, the reality is that many patients regularly experience delays that exceed this target.^2^ These delays can result in poorer treatment outcomes for patients,^3^ and in the cases of children and adolescents may exacerbate episodes of poor mental health in their adult years.^4^

Mental healthcare services, therefore, both seek to reduce waiting times and to temper the inimical effects of them.^5^ Technology has become an increasingly attractive solution for this,^5^ and patients themselves initiate help-seeking from AI while traditional mental health services are unavailable to them.^6^ In 2019, the NHS Topol review argued for the increased adoption of AI to balance expected demand for services and a decreasing supply of health professionals.^7^ However, the NHS has only recently started trialling the use of AI, with trials currently focusing on augmenting administrative tasks, rather than delivering therapy.^8^

Relational agents, often also referred to as conversational agents or chatbots, are an innovative AI tool in mental healthcare.^9^ These agents can appear in two forms: as either an application on the web and smart phones, or as social robots.^10^ Relational agents function in a variety of ways but most modern versions are increasingly large language models, trained on a plethora of text in order to generate one word at a time, until they produce grammatically coherent textual responses, reactions or instruction following.^11^ It is important to note that the responses that modern relational agents provide are probabilistic—they are likely sequences based on the training data that were fed to them.^12^

Relational agents are AI that specifically seek to build productive long-term relationships with patients,^13^ and they can be scalable interventions.^14 15^ Research indicates that relational agents can be effective and provide valuable support for mental health problems,^16^ but their effectiveness varies based on several factors, such as design, patient beliefs or the specific needs of individual patients.^17^ Relational agents, therefore, have the potential to expand mental healthcare, augmenting traditional therapy, but they need to be designed carefully to ensure safety, quality and a human element.^18^

One pressing area for relational agent design revolves around self-disclosure. Self-disclosure can be defined as the process of voluntarily sharing personal information about oneself with another, which the recipient is unlikely to know or discover from other sources.^19^ Individuals seek self-disclosure, as self-disclosure can create deeper bonds with and elicit help.^20^ Many seek therapy because they cannot self-disclose to others in the first instance.^21^ Of course, self-disclosure is not a monolith and so what, how and why people self-disclose varies.^22^ In the context of therapy, self-disclosure can create more intimate relationships between therapists and patients, contributing to the overall effectiveness of therapy.^23^ Therapist self-disclosure can enhance mutual trust and commitment.^24^ Patient self-disclosure can increase psychological resilience and allow patients to reframe their thoughts.^25^

Relational agents could therefore serve as the recipients of self-disclosure where traditional therapies are unavailable. One key benefit of relational agents is the anonymity and reduced fear of stigma that they afford to patients: individuals are often afraid that important but sensitive revelations such as sexual orientation could result in rejection and repercussions, for example, employment termination.^26 27^ While the evidence base for the general efficacy of relational agents is maturing, self-disclosure to relational agents is underexplored and no prior synthesis exists. We are therefore embarking on a comprehensive scoping review, which allows us the flexibility to explore qualitative, quantitative and mixed-method studies as well as explore multiple research questions to guide future research.

## Methods and analyses

### Aims and research questions

The scoping review aims to map literature on self-disclosure and relational agents for mental health. Our primary research question is as follows:

- To what degree do people disclose to relational agents in comparison to comparative interventions?

Our secondary research questions are:

- What factors increase self-disclosure to relational agents?
- What are the effects of agent self-disclosure on people?
- What are the effects of people’s self-disclosure on their mental health outcomes?

Our work will follow guidance by the Joanna Briggs Institute^28^ and the Preferred Reporting Items for Systematic Reviews and Meta-Analyses (PRISMA) Extension for Scoping Reviews.^29^

### Search strategy

Our review will systematically search 9 databases: Ovid Medline, Ovid Embase, Ovid Emcare, Ovid PsycINFO, Ovid Global Health, EBSCO CINAHL, Scopus, Web of Science and ProQuest Dissertations & Theses Global. We will also search, that is, EE Xplore and ACM Digital Library, and we will hand search relevant journals and the bibliographies of included studies. We will attach our piloted and final, full search strategy in Ovid Medline to this protocol (online supplemental file 1).

### Inclusion criteria

Our search will focus on peer-reviewed journal articles, Master’s and PhD theses, as well as full-length conference papers published in English. There will be no restrictions on publication dates.

#### Population

Any population irrespective of age, gender, ethnicity or health status will be included. There are no exclusions regarding study populations.

#### Intervention

Studies that administer any type of relational agents will be eligible. Relational agents could appear in a purely digital form, for example, a software programme, or they can appear with dedicated hardware, for example, social robots. We will not exclude any interventions.

#### Study design

We will include qualitative, quantitative and mixed-method studies. Randomised controlled trials (including quasi-RCTs and pilot RCTs), and non-randomised studies of interventions will be eligible. We will include studies focusing on all potential comparators, for example, relational agents without self-disclosure, waiting lists and human counsellors. We will not exclude studies due to the presence or absence of long-term follow-up, but we will exclude protocols, narrative reviews, systematic reviews, opinion pieces and theoretical papers without primary data.

#### Setting

Studies in any setting (eg, care homes, schools, hospitals) will be eligible.

### Screening and data extraction

We will de-duplicate results and complete screening in Covidence, which allows effective coordination among team members. Three review authors will independently screen titles and abstracts in duplicate. Disagreements will be resolved by a lead researcher. Full texts will be uploaded via EndNote (chosen for superior automatic download performance during trials) or manually where needed. Full-text screening will follow the same process, with disagreements resolved through discussion and adjudication by the lead researcher. Training will be provided before each screening stage to promote inter-rater agreement, measured via Cohen’s kappa.^30^ We will aim for ≥75% agreement, with iterative monitoring, further training and a formal inclusion/exclusion codebook as needed. Data will be extracted independently by three reviewers in duplicate using a structured extraction form, which we have attached (online supplemental file 2).

### Data analysis

We will perform a narrative synthesis of the findings following guidance of Popay *et al*,^31^ including tabulations and charts where appropriate. We expect included studies to primarily report quantitative outcomes, for example, depression or self-disclosure. Measures of these outcomes will vary, for example, while some researchers used standardised scales to report self-disclosure, Ho *et al*^32^ developed their own classification system.

Additionally, we will explore descriptive statistics of quantitative results. We expect to provide measures of frequency and central tendency, for example, frequency of studies where there was increased disclosure to agents.

### Timelines

The review will start in December 2024 and complete in August 2025.

## Ethics and dissemination

The scoping review does not require ethical review due to its use of publicly available data. It does not collect data requiring consent from participants. The findings of the scoping review will be published in a peer-reviewed journal, and we will present them at academic conferences.

## Supplementary material

10.1136/bmjopen-2025-100613 online supplemental file 1

10.1136/bmjopen-2025-100613 online supplemental file 2
