# Supplementary material for: Self-disclosure and relational agents for mental health: a scoping review protocol
Source: BMJ Open. 2025 Aug 24;15(8):e100613. doi: 10.1136/bmjopen-2025-100613 (PMC12382548; doi:10.1136/bmjopen-2025-100613)
Supplement: online supplemental file 1 [file bmjopen-15-8-s001.docx]

Ovid MEDLINE(R) ALL <1946 to November 08, 2024>

1 exp Artificial Intelligence/ 213688

2 User-Computer Interface/ 40030

3 Robotics/ 29860

4 (conversation* agent* or relation* agent* or digital* human? or computer* agent* or chat bot* or chat-bot* or chatbot* or companion* agent* or virtual agent* or intelligent agent* or personal* agent* or voice agent* or digital coach* or digital* assistant* or voice assistant* or robot* or avatar* or dialog system* or digital people or natural language interface* or human-agent* or woebot or replica or mylo or wysa or gabby or eSMART-MH or help4mood or sabori or tanya or aibo or paro or irobiQ or cafero or virtual coach* or chat agent* or virtual human* or conversation* interface* or digital person? or artificial intelligence or AI).tw. 183990

5 or/1-4 375383

6 Self-Disclosure/ 7387

7 Disclosure/ 15928

8 (disclosure* or divulg*).ti. 8173

9 (self-disclos* or self disclos* or self-revelation* or self revelation* or self reveal* self-reveal* or personal reveal* or self-express* or self express* or personal express* or self-divulg* or self divulg* or personal divulge* or self shar* or self-shar* or personal shar*).tw. 3069

10 or/6-9 28807

11 5 and 10 177

12 limit 11 to english language 176
