# Supplementary material for: Self-disclosure and relational agents for mental health: a scoping review protocol
Source: BMJ Open. 2025 Aug 24;15(8):e100613. doi: 10.1136/bmjopen-2025-100613 (PMC12382548; doi:10.1136/bmjopen-2025-100613)
Supplement: online supplemental file 2 [file bmjopen-15-8-s002.docx]

**# Data Extraction Template**

**## General**

- ****Title****

- ****Last name of lead author****

- ****Country in which the study was conducted****:

- United States

- UK

- South Korea

- Japan

- China

- Other

- ****Year of publication****

- ****Type****:

- Peer-reviewed journal article

- Theses

- Conference article

- Other

- ****Number of studies in the paper****:

- Single

- Multiple

**## Characteristics of Included Studies**

**### Methods**

- ****Study strategy****:

- Quantitative

- Qualitative

- Mixed

- ****Study design****:

- Randomised controlled trial

- Non-randomised experimental study

- Cross-sectional study

- Other

- ****Does a comparator exist?****:

- Yes

- No

- ****Was agent self-disclosure manipulated?****:

- Yes

- No

- ****Manipulation****

- ****Study procedure****

**### Participants**

- ****Population description****

- ****Total number of participants****

- ****Mean age****

- ****Age range****

- ****Percentage female****

- ****Percentage ethnic minority****

**### Relational Agent**

- ****Name of relational agent****

- ****Description of relational agent****

- ****Nature of agent****:

- Autonomous

- Semi-autonomous

- Wizard-of-Oz

**## Results**

- ****Study topic****:

- Only about mental health

- Mental health + some other topics

- ****Was patient self-disclosure measured as an outcome?****:

- Yes

- No

- ****Outcome measured****

- ****Self-disclosure measurement tool****

- ****Number of measurements****:

- At one point in time

- Several points

**## Summary**

- ****Summary****

- ****Any comments?****
