# Supplementary material for: Self-disclosure and relational agents for mental health: a scoping review protocol
Source: BMJ Open. 2025 Aug 24;15(8):e100613. doi: 10.1136/bmjopen-2025-100613 (PMC12382548; doi:10.1136/bmjopen-2025-100613)
Supplement: Supplementary file 3 [file bmjopen-2025-100613.reviewer_comments.pdf]

PEER REVIEW HISTORY

BMJ Open publishes all reviews undertaken for accepted manuscripts. Reviewers are asked to complete a checklist review form and are provided with free text boxes to elaborate on their assessment. These free text comments are reproduced below.

ARTICLE DETAILS

Title (Provisional)

Self-disclosure and relational agents for mental health: a scoping review protocol

Authors

Sha, Sia; Loveys, Kate; Francis, Isla; Ji, Eric; Lyu, Leo; Krpan, Dario; Galizzi, Matteo Maria; Taylor, Mark

---

VERSION 1 - REVIEW

Reviewer 1

Name Bhavsar, Vishal

Affiliation Institute of Psychiatry Department of Health Service and Population Research, Department of Health Services and Population Research

Date 26-Mar-2025

COI None

Thank you. This looks worthwhile.

In the abstract, please define relational agents and ideally include a description of for a lay(ish) audience- e.g. referring to "chatbots", the typical interface over a net-enabled device, involving a relation i.e. a prompt-response, or question and answer format.

Can you specific a research objective or a research question in the abstract.

Intro looks ok. You might helpfully include information on any policy responses to NHS workforce needs, including in relation to AI.

Include further top-level information on how relational agents work(for a non-expert scientifically informed audience).

Some of your more leading statements- e.g. there is a deep desire to self-disclose; the benefit of RAs to stigma- could do with some description of evidence in the text(beyond just including citations.

Reviewer 2

Name **Gill, Shané**

Affiliation **Thomas Jefferson University, Family & Community Medicine**

Date **15-Apr-2025**

COI **None**

General: The choice of topic is of interest and appears to be relevant to the field. The authors write with clarity overall and the protocol is succinct. There appears to be an understanding of the appropriate framework and application of this framework to explore existing gaps in the field as it pertains to this topic. There are however, immense concerns about the use of the correct framework used to develop the research question, background literature and the needs statement, methodology, and chosen approach for data analysis. The protocol can be significantly improved by addressing the items below and reiterating the necessity of a scoping review.

Title: Per JBI guidelines and the PRISMA-ScR framework, the title must specify that this is a scoping review protocol. For example, "Self-disclosure and Relational Agents for Mental Health: a Scoping Review Protocol." This submission is not a full review.

Introduction: The authors include one citation to support arguments proposed throughout the introduction. Additional literature to support assertions and propositions is necessary. For example, the authors state "relation agents seek to build productive long-term relationships with patients, and they are scalable interventions that are particularly suited to cognitive behavioural therapy..." (line 52-53) with no citations but use a followup statement asserting that relational agents are effective to support mental health problems. This is a poor transition between concepts. The authors would benefit from elaborating first on how the delays in access to mental health services has contributed to an uptake in relational agents (paragraph 1) because there is an abrupt transition between the structural factors that impact provisions of care in NHS and the emergence of relational agents which are being framed as a dual-solution-one that expands access to care and one that promotes engagement to improve health outcomes. Provide additional citations to support the information about contraindications of relational agents. It should be clear that despite known limitations (e.g., variations based on several factors, line 59), the benefits are imminent warranting further exploration. However, progress in therapy is heavily contingent upon clients' willingness to self-disclosure (this should be a transitional sentence). Paragraphs should include a summarizing sentence. Lastly, in the introduction, it is unclear why closing the gap between "which patients, topics, and conditions make disclosure to self-relation agents preferential" (lines 25-26) is critical and the necessity of a scoping review compared to other reviews is not stated explicitly. Adding two to three sentences on the use of the scoping review as the most appropriate method would strengthen this introduction.

Methods and Analyses

Aims and Research Questions: It is unclear which question is the primary research question. It would be appropriate to specify the primary research question and list the other questions in order of sequence. The researchers appear to be using the PICO framework (Population, Intervention, Comparison, and Outcome) which should be stated explicitly.

Search Strategy: Use of hand searching (mining) is an excellent strategy to increase

Inclusion Criteria: This is the first mention of the types of relational agents but there is no mention of these in the introduction. Blanket statements were made about the efficacy and relational agents in the introduction, however, a brief statement about the types should have been included. It would appear that mixed-methods studies would be appropriate based on the nature of the research questions.

Exclusion Criteria: The researchers would benefit from exploring this further. Studies with specific methods were excluded and design. Specify if there are comparative interventions that would be excluded and the rationale for example. If there are specific mental health outcomes that would be excluded (e.g., severe mental illness for which use of relational agents may be deemed inappropriate or there is not sufficient evidence or data yet available on persons with SMI), settings such as tele-health or remote for example in which use of this service is self-initiated. Again, these are considerations and may not be appropriate. It is to challenge to you to consider if there are certain criteria that should be excluded.

Screening and Data Extraction: The rationale for using Covidence and EndNote is unclear. EndNote is primarily used as a citation manager and is considered primitive as it pertains to its utility for screening and de-duplication. The second step in data analysis is full-text screening at which time, the researchers can manually search for articles that do not have the full text and include a URL or attach a PDF of the article. There is no information about the lead researcher (third reviewer) will document discrepancies, advise the team, and use this information to iteratively make revisions too criteria as needed and agreement on these revisions. Cohen's kappa should be cited. 75% agreement should be achieved. There is no information on modifications that would be made or additional training should the pilot be unsuccessful in achieving 75% agreement.

Data Analysis: Descriptive analysis is typically used in scoping reviews. This can include frequency counts, basic categorization of data (e.g., outcomes associated with relational agents and percent efficacy), and identifying key concepts and contexts for example. Similarly, qualitative content analysis is also used to identify patterns or themes that emerge in the data. Narrative analysis is used to understand meaning of experiences and how they are described which could be appropriate for qualitative studies or mixed-methods studies that include patient reports of efficacy or their experiences of relational agents. Consider the appropriateness of the proposed methods to answer the research question (primary question).

VERSION 1 - AUTHOR RESPONSE

Reviewer: 1
Dr. Vishal Bhavsar, Institute of Psychiatry Department of Health Service and Population Research
Comments to the Author:
- Thank you. This looks worthwhile. In the abstract, please define relational agents and ideally include a description of for a lay(ish) audience- e.g. referring to "chatbots", the typical interface over a net-enabled device, involving a relation i.e. a prompt-response, or question and answer format. This is a good point. Done (line 9-13).
- Can you specific a reserch objective or a research question in the abstract. Intro looks ok. Done (line 12-13).
- You might helpfully include information on any policy responses to NHS workforce needs, including in relation to AI. A good suggestion. Done (line 58-61).
- Include further top-level information on how relational agents work(for a non-expert scientifically informed audience). Yes. Helpful. Done (line 65-70).
- Some of your more leading statements- e.g. there is a deep desire to self-disclose; the benefit of RAs to stigma- could do with some description of evidence in the text(beyond just including citations. Thanks for this. We adjusted several ones (e.g. line 83-84 and 94-95).

Reviewer: 2
Dr. Shané Gill, Thomas Jefferson University
Comments to the Author:
- General: The choice of topic is of interest and appears to be relevant to the field. The authors write with clarity overall and the protocol is succinct. There appears to be an understanding of the appropriate framework and application of this framework to explore existing gaps in the field as it pertains to this topic. There are however, immense concerns about the use of the correct framework used to develop the research question, background literature and the needs statement, methodology, and chosen approach for data analysis. The protocol can be significantly improved by addressing the items below and reiterating the necessity of a scoping review.Title: Per JBI guidelines and the PRISMA-ScR framework, the title must specify that this is a scoping review protocol. For example, "Self-disclosure and Relational Agents for Mental Health: a Scoping Review Protocol." This submission is not a full review. Done (line 1)!

- Introduction: The authors include one citation to support arguments proposed throughout the introduction. Additional literature to support assertions and propositions is necessary. For example, the authors state "relation agents seek to build productive long-term relationships with patients, and they are scalable interventions that are particularly suited to cognitive behavioural therapy..."

(line 52-53) with no citations but use a followup statement asserting that relational agents are effective to support mental health problems. This is a poor transition between concepts. We have provided references now. Thank you (line 72-3).

- The authors would benefit from elaborating first on how the delays in access to mental health services has contributed to an uptake in relational agents (paragraph 1) because there is an abrupt transition between the structural factors that impact provisions of care in NHS and the emergence of relational agents which are being framed as a dual-solution-one that expands access to care and one that promotes engagement to improve health outcomes. Provide additional citations to support the information about contraindications of relational agents. It should be clear that despite known limitations (e.g., variations based on several factors, line 59), the benefits are imminent warranting further exploration. However, progress in therapy is heavily contingent upon clients' willingness to self-disclosure (this should be a transitional sentence). Paragraphs should include a summarizing sentence. Lastly, in the introduction, it is unclear why closing the gap between "which patients, topics, and conditions make disclosure to self-relation agents preferential" (lines 25-26) is critical and the necessity of a scoping review compared to other reviews is not stated explicitly. Adding two to three sentences on the use of the scoping review as the most appropriate method would strengthen this introduction. These are great suggestions. We have now rewritten several sections across the introduction (e.g. line 92-100).

- Methods and Analyses
Aims and Research Questions: It is unclear which question is the primary research question. It would be appropriate to specify the primary research question and list the other questions in order of sequence. The researchers appear to be using the PICO framework (Population, Intervention, Comparison, and Outcome) which should be stated explicitly. Thank you for this. We adjusted the objectives as suggested, but we do not follow PICO (line 135-40).

- Search Strategy: Use of hand searching (mining) is an excellent strategy to increase (Comment trails off …)

-Inclusion Criteria: This is the first mention of the types of relational agents but there is no mention of these in the introduction. Blanket statements were made about the efficacy and relational agents in the introduction, however, a brief statement about the types should have been included. It would appear that mixed-methods studies would be appropriate based on the nature of the research questions. We have included type in the introduction (line 64-5) and emphasised that mixed-method studies are included (line 171).

Exclusion Criteria: The researchers would benefit from exploring this further. Studies with specific methods were excluded and design. Specify if there are comparative interventions that would be excluded and the rationale for example. If there are specific mental health outcomes that would be excluded (e.g., severe mental illness for which use of relational agents may be deemed inappropriate or there is not sufficient evidence or data yet available on persons with SMI), settings such as tele-health or remote for example in which use of this service is self-initiated. Again, these are considerations and may not be appropriate. It is to challenge to you to consider if there are certain criteria that should be excluded. There are no exclusions and we have made this clearer (line 163 and 168). Our aim is to be comprehensive in this review and inform the reader of the entire research landscape. For example, if there isn't sufficient data on severe mental illness, that would be a valuable finding.

Screening and Data Extraction: The rationale for using Covidence and EndNote is unclear. EndNote is primarily used as a citation manager and is considered primitive as it pertains to its utility for screening and de-duplication. The second step in data analysis is full-text screening at which time, the researchers can manually search for articles that do not have the full text and include a URL or attach a PDF of the article. There is no information about the lead researcher (third reviewer) will document discrepancies, advise the team, and use this information to iteratively make revisions too criteria as needed and agreement on these revisions. Cohen's kappa should be cited. 75% agreement should be achieved. There is no information on modifications that would be made or additional training should the pilot be unsuccessful in achieving 75% agreement. We have now adjusted this section to make it clearer (line 195-200). Thank you.

Data Analysis: Descriptive analysis is typically used in scoping reviews. This can include frequency counts, basic categorization of data (e.g., outcomes associated with relational agents and percent efficacy), and identifying key concepts and contexts for example. Similarly, qualitative content analysis is also used to identify patterns or themes that emerge in the data. Narrative analysis is used to understand meaning of experiences and how they are described which could be appropriate for qualitative studies or mixed-methods studies that include patient reports of efficacy or their experiences of relational agents. Consider the appropriateness of the proposed methods to answer the research question (primary question). Thank are performing a narrative synthesis of results. Thank you for this. We have included descriptive analysis, which is a great suggestion (line 214-216).
